# Supplementary material for: Bacterial killing by complement requires membrane attack complex formation via surface‐bound C5 convertases
Source: EMBO J. 2019 Jan 14;38(4):e99852. doi: 10.15252/embj.201899852 (PMC6376327; doi:10.15252/embj.201899852)
Supplement: Supplementary file 1 — Expanded View Figures PDF [file EMBJ-38-e99852-s001.pdf]

Expanded View Figures

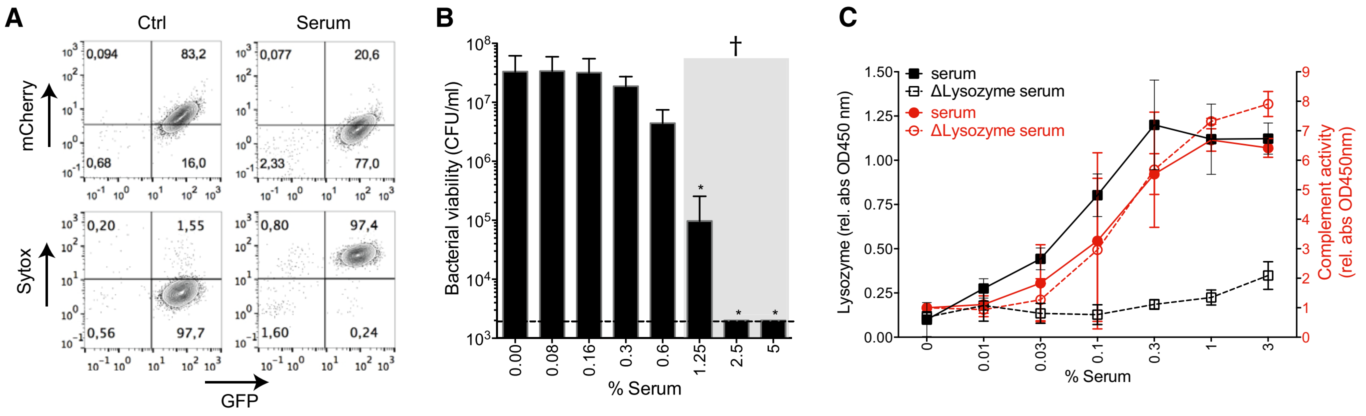

Figure EV1. Serum-induced inner membrane damage is essential for bacterial killing.

A Representative flow cytometry plots of $_{peri}$mCherry/$_{cyto}$GFP *E. coli* after 30 min of exposure to buffer or 10% human serum.

B Bacterial viability (via colony enumeration on agar plates) of $_{peri}$mCherry/$_{cyto}$GFP *E. coli* exposed to a concentration range of serum (samples identical to Fig 1C).

C Successful depletion of serum from lysozyme (lysozyme-specific ELISA; black line), but sustained complement activity (CH50; red).

Data information: (B, C) Data represent mean ± SD of 3 independent experiments. (B) Statistical analysis was done using a ratio paired two-tailed *t*-test and displayed only when significant as *$P \leq 0.05$.

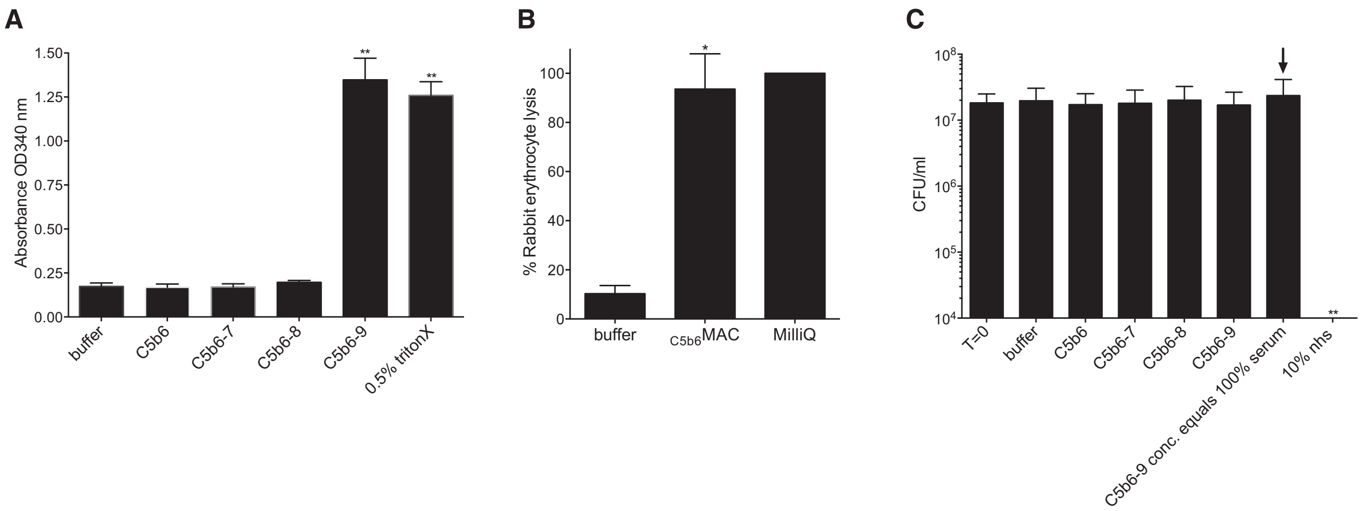

Figure EV2. MAC assembled from purified C5b6 lacks bactericidal activity.

A Lysis of liposomes after exposure to preassembled C5b6 with or without C7, C8, and C9. Calcein release from liposomes was determined by measuring absorbance at OD340 nm; 0.5% Triton X-100 was used as a positive control.

B Percentage lysis of rabbit erythrocytes exposed to buffer or $_{C5b6}$MAC, compared to Milli-Q (MQ) water as control (set at 100% lysis).

C Killing of *E. coli* MG1655 after exposure to preassembled C5b6 with or without C7, C8, and C9 (at concentrations similar to (B) or at concentrations exceeding those of 100% serum (highlighted by an arrow); ± 100 nM C5b6, 600 nM C7, 350 nM C8, 900 nM C9).

Data information: Data represent mean ± SD of 3 independent experiments. Statistical analysis was done using a ratio paired two-tailed *t*-test and displayed only when significant as *$P \leq 0.05$ or **$P \leq 0.01$.

© 2019 The Authors

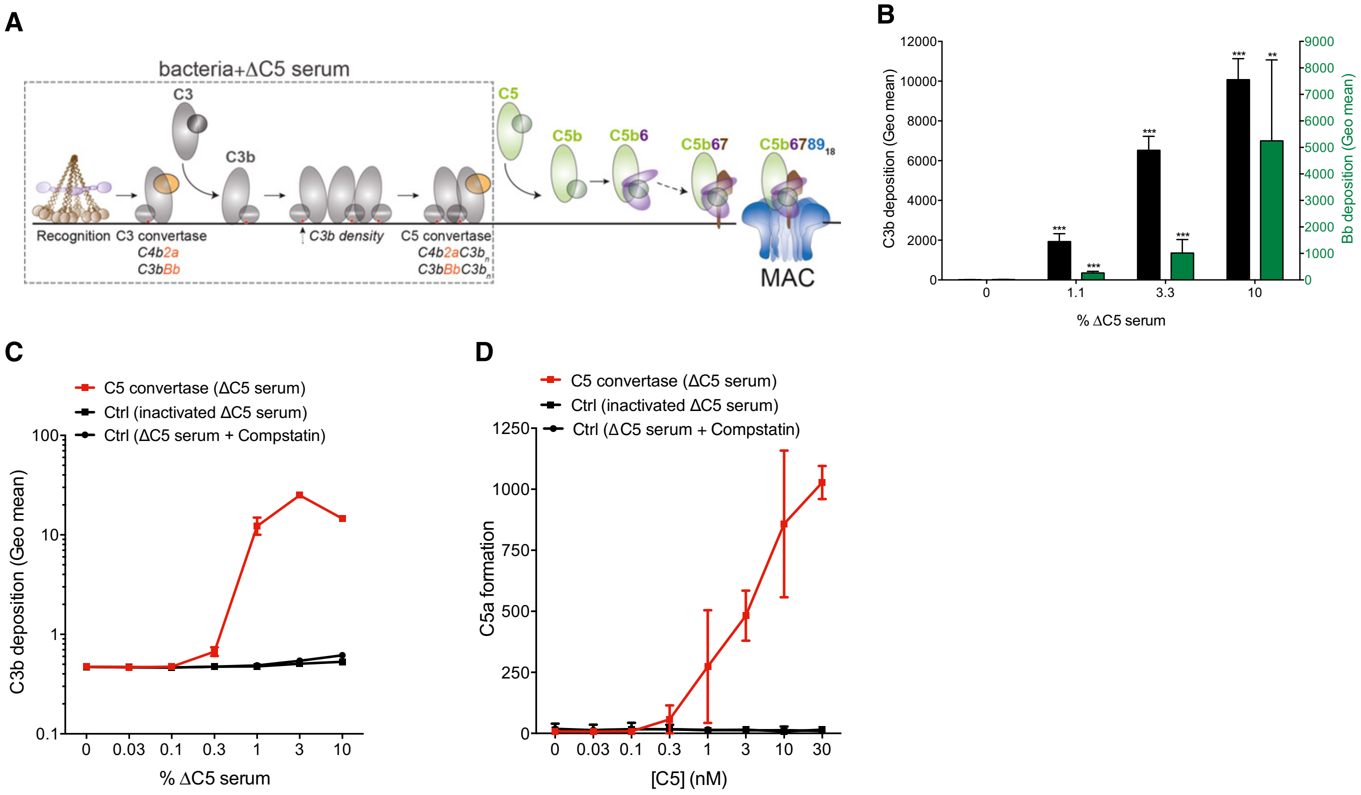

Figure EV3. Successful labeling of the bacterial surface with C5 convertases.

A Schematic overview of complement activation and MAC formation on the bacterial membrane. Different recognition pathways (classical, lectin, and alternative) generate C3 convertases (C4b2a in the classical/lectin pathway, C3bBb in the alternative pathway) on the target cell surface that cleave the major complement protein C3 into C3b. C3b covalently attaches to the cell surface via a reactive thioester. At high C3b densities, C3 convertases associate with deposited C3b to form C5 convertases (C4b2aC3b in the classical/lectin pathway, C3bBbC3b in the alternative pathway). The C5 convertase then catalyzes conversion of C5 into C5a and C5b. C5b triggers MAC formation by sequential binding to C6, C7, C8, and multiple copies of C9.

B Incubation of *E. coli* MG1655 with a concentration range of C5-depleted serum (ΔC5 serum) results in surface labeling with alternative pathway convertases (C3bBbC3b, evidence by flow cytometry analysis of surface-bound C3b and Bb).

C, D Successful labeling of *E. coli* with C5 convertases. (C) *E. coli* MG1655 was pre-incubated with 10% ΔC5 serum (labeled as: C5 convertase), heat-inactivated ΔC5 serum (Ctrl), or ΔC5 serum supplemented with 5 μM compstatin (Ctrl). After washing, C3b deposition was measured by flow cytometry. (D) Bacteria were labeled as described in (C) and (after washing) incubated with a concentration range of C5. Release of C5a into supernatants was measured by a calcium flux-based assay (Bestebroer *et al*, 2010).

Data information: (B–D) Data represent mean ± SD of 3 independent experiments. (B) Statistical analysis was done using a ratio paired two-tailed *t*-test and displayed only when significant as $^{**}P \leq 0.01$ or $^{***}P \leq 0.001$.

© 2019 The Authors

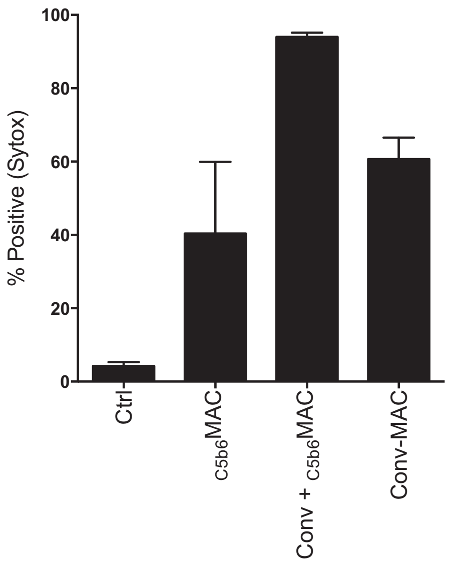

Figure EV4. Local assembly of C5b6 by surface-bound C5 convertases is not essential to lyse human HAP1 cells.

Percentage lysis of non-opsonized or convertase-labeled HAP1 cells exposed to 100 nM C5b6 ($_{C5b6}$MAC) or C5 and C6 (Conv-MAC) in the presence of 100 nM C7, C8, and C9. Data represent mean ± SD of 2 independent experiments.

 © 2019 The Authors

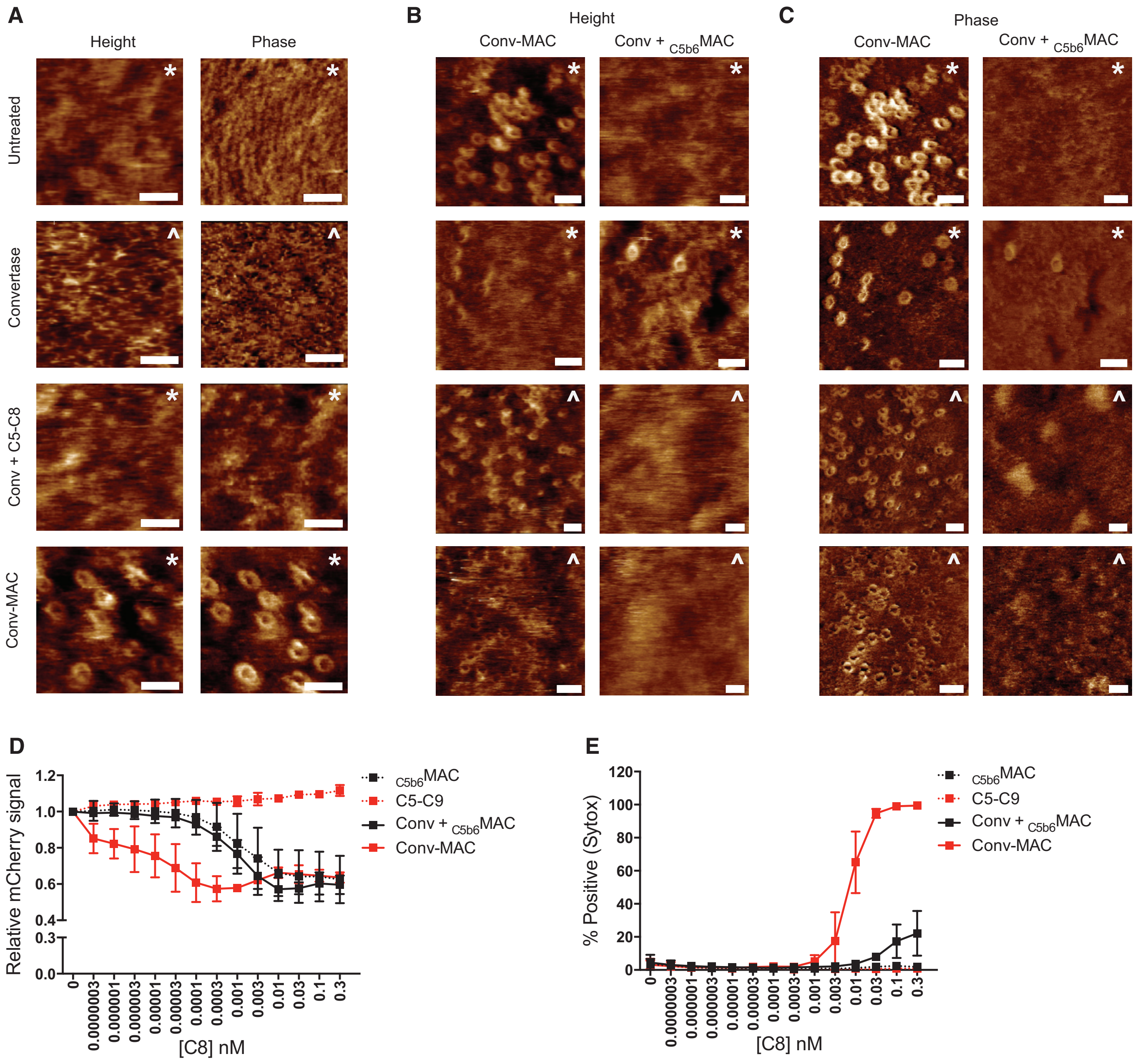

Figure EV5. Local assembly of C5b6 is required for stable insertion of MAC pores and efficient outer membrane damage.

A Atomic force microscopy height (left) and phase (right) images of *E. coli* MG1655 incubated with buffer (untreated), convertases, and convertases plus either C5-C8 (Conv + C5-C8) or C5-C9 (Conv-MAC). Scale bars: 50 nm. Height scales: 5 nm (untreated), 9 nm (convertase), and 6 nm (conv + C5-C8/MAC).

B, C Atomic force microscopy height (B) and phase (C) images of convertase-labeled *E. coli* MG1655 exposed to either C5 and C6 or preassembled C5b6 (Conv-MAC versus Conv + $_{C5b6}$MAC) in the presence of C7-C9, FB, and FD. Data shown correspond to four separate experiments; in each experiment, Conv-MAC and Conv + $_{C5b6}$MAC were compared directly. The upper images of (B and C) are also presented in Fig 8D. Scale bars: 50 nm. Height scales: 15 nm.

D, E (D) Outer membrane damage (mCherry) and (E) inner membrane damage (% Sytox positive) of non-opsonized or convertase-labeled bacteria incubated with 10 nM of C5 and C6 or C5b6 in the presence of 10 nM C7. After washing, a concentration range of C8 and 100 nM C9 was added. Data represent mean ± SD of 3 independent experiments.

Data information: (A–C) Experiments were carried out using either the Poly-L-lysine (*) or Cell-Tak (^) immobilization protocols, in 10 mM PB or PBS, respectively.

© 2019 The Authors
